# Bleeding in minimally invasive versus conventional aortic valve replacement

**DOI:** 10.1186/s13019-024-02667-1

**Published:** 2024-06-21

**Authors:** Sorosh Bratt, Axel Dimberg, Mikael Kastengren, Robert D. Lilford, Peter Svenarud, Ulrik Sartipy, Anders Franco-Cereceda, Magnus Dalén

**Affiliations:** 1 Department of Cardiothoracic Surgery, Karolinska University Hospital, Stockholm, SE-17176 Sweden; 2 Department of Molecular Medicine and Surgery, Karolinska Institutet, Stockholm, Sweden; 3 Department of Cardiology, Karolinska University Hospital, Stockholm, Sweden

**Keywords:** Aortic valve surgery, Bleeding, Minimally invasive surgery, Ministernotomy

## Abstract

**Background:**

Observational studies have shown reduced perioperative bleeding in patients undergoing minimally invasive, compared with full sternotomy, aortic valve replacement. Data from randomized trials are conflicting.

**Methods:**

This was a Swedish single center study where adult patients with aortic stenosis, 100 patients were randomly assigned in a 1:1 ratio to undergo either minimally invasive (ministernotomy) or full sternotomy aortic valve replacement. The primary outcome was severe or massive bleeding defined by the Universal Definition of Perioperative Bleeding in adult cardiac surgery (UDPB). Secondary outcomes included blood product transfusions, chest tube output, re-exploration for bleeding, and several other clinically relevant events.

**Results:**

Out of 100 patients, three patients randomized to ministernotomy were intraoperatively converted to full sternotomy (none was bleeding-related). Three patients (6%) in the full sternotomy group and 3 patients (6%) in the ministernotomy group suffered severe or massive postoperative bleeding according to the UDPB definition (*p* = 1.00). Mean chest tube output during the first 12 postoperative hours was 350 (standard deviation (SD) 220) ml in the full sternotomy group and 270 (SD 190) ml in the ministernotomy group (*p* = 0.08). 28% of patients in the full sternotomy group and 36% of patients in the ministernotomy group received at least one packed red blood cells transfusion (*p* = 0.39). Two patients in each group (4%) underwent re-exploration for bleeding.

**Conclusions:**

Minimally invasive aortic valve replacement did not result in less bleeding-related outcomes compared to full sternotomy.

**Clinical Trial Registration:**

http://www.clinicaltrials.gov. Unique identifier: NCT02272621.

**Supplementary Information:**

The online version contains supplementary material available at 10.1186/s13019-024-02667-1.

## Introduction

In aortic valve replacement (AVR), surgical access through a partial upper ministernotomy presents a less invasive alternative to a standard full sternotomy [1, 2]. Possible reduction of bleeding is one of several desired benefits motivating the transition towards minimally invasive surgery [3, 4]. Avoiding excessive bleeding is of paramount importance in cardiac surgery since blood loss, transfusions, and re-exploration for bleeding have a well-established association to other postoperative complications and mortality [5, 6].

Adoption of the ministernotomy approach in AVR has increased since large retrospective and propensity matched series have suggested improved overall outcomes including reduced mortality, rate of blood transfusions and postoperative length of stay, and the method is reportedly used in 15% of cases of aortic valve replacement in the United States [2, 7, 8]. However, conflicting results regarding bleeding-related complications are emerging from randomized studies, and the two largest randomized trials comparing ministernotomy showed no difference in their primary outcomes of red blood cell transfusion rates and hospital stay [9, 10].

We performed a single center, nonblinded, randomized controlled trial to analyze differences in severe perioperative bleeding in AVR for aortic valve stenosis performed with ministernotomy versus full sternotomy. The hypothesis was that minimally invasive AVR would result in less bleeding complications.

## Methods

### Study design

This was a single-center, randomized controlled parallel-group trial using 1:1 randomization. Regional Human Research Ethics Committee approval and informed patient consent was obtained. The study was registered at ClinicalTrials.gov (http://www.clinicaltrials.gov; Unique identifier: NCT02272621). Study design, data collection, and manuscript writing were performed by the authors with no contribution from sponsors. The reporting follows the recommendations in the CONSORT 2010 statement (Consolidated Standards of Reporting Trials) [11].

### Study population and randomization

Eligible participants were patients with symptomatic severe aortic valve stenosis, 18 years or older, scheduled for isolated AVR at Karolinska University Hospital in Stockholm, Sweden. Exclusion criteria were left ventricular ejection fraction < 45%, previous cardiac surgery, urgent surgery, or participation in another clinical study. Randomization was done between November 2013 and August 2020. The first 40 included patients were intially included in a study regarding right ventricular function in ministernotomy versus full sternotomy AVR, which has previously been published [12, 13]. In this previously published study, the sample size was based on hypothesized differences in right ventricular function and did not aim to compare the two surgical methods regarding other postoperative outcomes. We therefore increased the sample size to further investigate bleeding-related outcomes. The choice to include 100 patients was pragmatic, as it was achievable within a reasonable time frame and that a clinically significant difference in bleeding-related outcomes would manifest with this number. No additional sample size calculation was done for the current study.

A research nurse was in charge for enrolling, and asked patients who met inclusion criteria and none of the exclusion criteria if they were willing to participate in the study. The investigating surgeon generated the random allocation sequence and randomized the patient before being admitted to the hospital for surgery. Patients were randomly assigned 1:1 to either ministernotomy or full sternotomy AVR. A blinded envelope system using sequentially numbered containers was used to randomize patients to intervention without blocking. After randomization, the patient was informed per telephone whether AVR should be performed with ministernotomy or full sternotomy. The surgeon and the physician performing the follow-up were not blinded to study group assignment. Four surgeons with experience in ministernotomy AVR operated patients in both study groups and additional four surgeons operated patients in the full sternotomy group. All participating surgeons were consultants.

### Study endpoint

Severe or massive bleeding, as defined by the “Universal Definition of Perioperative Bleeding in adult cardiac surgery” (UDPB), was selected as a clinically relevant composite primary outcome [14]. According to the UDPB definitions, at least one of the following criteria must be fulfilled to attain the composite primary outcome: Delayed sternal closure (i.e. leaving the operating room with open chest due to bleeding), postoperative bleeding more than 1000 ml, transfusion of > 4 packed red blood cell or plasma, administration of recombinant activated factor VII or re-exploration for bleeding. Secondary outcomes included all blood product transfusions, chest tube output 12 h, re-exploration for bleeding, death from any cause, major adverse cardiac event (defined as defined as 30-day mortality, perioperative stroke, or perioperative myocardial infarction, defined as signs of ischemia in at least 2 of the parameters electrocardiogram, echocardiography or creatine kinase-MB more than 50 micrograms per liter), duration of intensive care unit stay and total length of hospital stay.

### Aortic valve replacement

In patients undergoing ministernotomy, a 6 cm midline skin incision was made over the upper part of the sternum. A partial J-shaped ministernotomy to the right third intercostal space was performed. A cranial small partial pericardial incision was made anterior to the ascending aorta. Cardiopulmonary bypass was established with central arterial and peripheral venous cannulation through the femoral vein. The pericardial opening was closed at the end of the procedure.

In patients undergoing full sternotomy, the skin incision was made between the suprasternal notch and the xiphoid process and the sternum divided in the midline. The pericardium was opened in full in the midline and left open at the end of surgery. Cardiopulmonary bypass was established with central arterial and venous cannulation.

In both ministernotomy and full sternotomy procedures, the left ventricle was decompressed through a right upper pulmonary vein drainage, the ascending aorta was incised, the aortic valve excised, and the annulus completely decalcified. Mechanical and bioprosthetic aortic valves were used. Temporary pacemaker wires were placed on the right ventricle and the sternum was closed with wires. Chest tubes were placed in the pericardium/mediastinum and opened pleural cavities, which was according to the surgeon’s preferences. During the study period there were no changes in surgical technique or perioperative care, or surgeons performing the procedures.

### Antithrombotic and hemorrhage treatment

Warfarin and non-vitamin K oral anticoagulants were paused at least 3 days, and clopidogrel at least 5 days before surgery. Acetylsalicylic acid treatment was uninterrupted. A bolus dose of 2 g of tranexamic acid was given before skin incision followed by infusion of 1 g/hour during surgery. Before cannulation, heparin was administered (400 IU/kg; activated coagulation time (ACT) maintained > 480 s), and after weaning from cardiopulmonary bypass, heparin was neutralized with protamine at a 1:1 ratio. Postoperative low molecular weight heparin was administered at a dose of 5000 units (or 2500 units for patients weighing less than 50 kg) on the evening the day after surgery, unless contraindicated due to ongoing bleeding.

Chest tube output was registered hourly postoperatively. The ACT was analyzed if chest drainage was more than 200 ml during the first postoperative hour or more than 100 ml/h after the first postoperative hour. An additional 50 mg of protamine was administered if ACT > 140 s. If excessive bleeding persisted, coagulation status including platelet count, prothrombin time, activated partial thromboplastin time, fibrinogen, and antithrombin III was performed. Results from the laboratory tests guided administration of coagulation factor concentrates. Packed red blood cells were transfused if the postoperative hemoglobin level was less than 70 g/l and repeated if indicated by subsequent testing. Hemodynamic instability in combination with bleeding of more than 300 mL/h, or more than 200 mL/h during 2 consecutive hours, despite amendment of coagulation disorders, were indications for surgical re-exploration. Chest tubes were removed the day after surgery if bleeding was less than 30 ml/h for 3 consecutive hours. All decisions regarding re-exploration for bleeding and administration of transfusions were ultimately made at the discretion of the surgeon.

### Data collection

Clinical data including bleeding-related data were registered by a research nurse. All data was collected prospectively.

### Statistical methods

Patients were analyzed according to intention to treat, i.e. patients intraoperatively converted from ministernotomy to full sternotomy were analyzed in the ministernotomy group. Variables were described using frequencies and percentages for categorical variables and means and standard deviations (SD) or medians and interquartile range (quartile [Q] 1, Q3) for continuous variables. Continuous variables were compared using independent samples t-test or Wilcoxon sum-rank test. Categorical or binary variables were compared using Pearson’s chi-squared test. A two-sided p-value of less than 0.05 was considered to indicate statistical significance. Data management and statistical analyses were performed using Stata 17.1 (Stata Corp, College Station, TX, USA).

## Results

### Study population

**Fig. 1:**
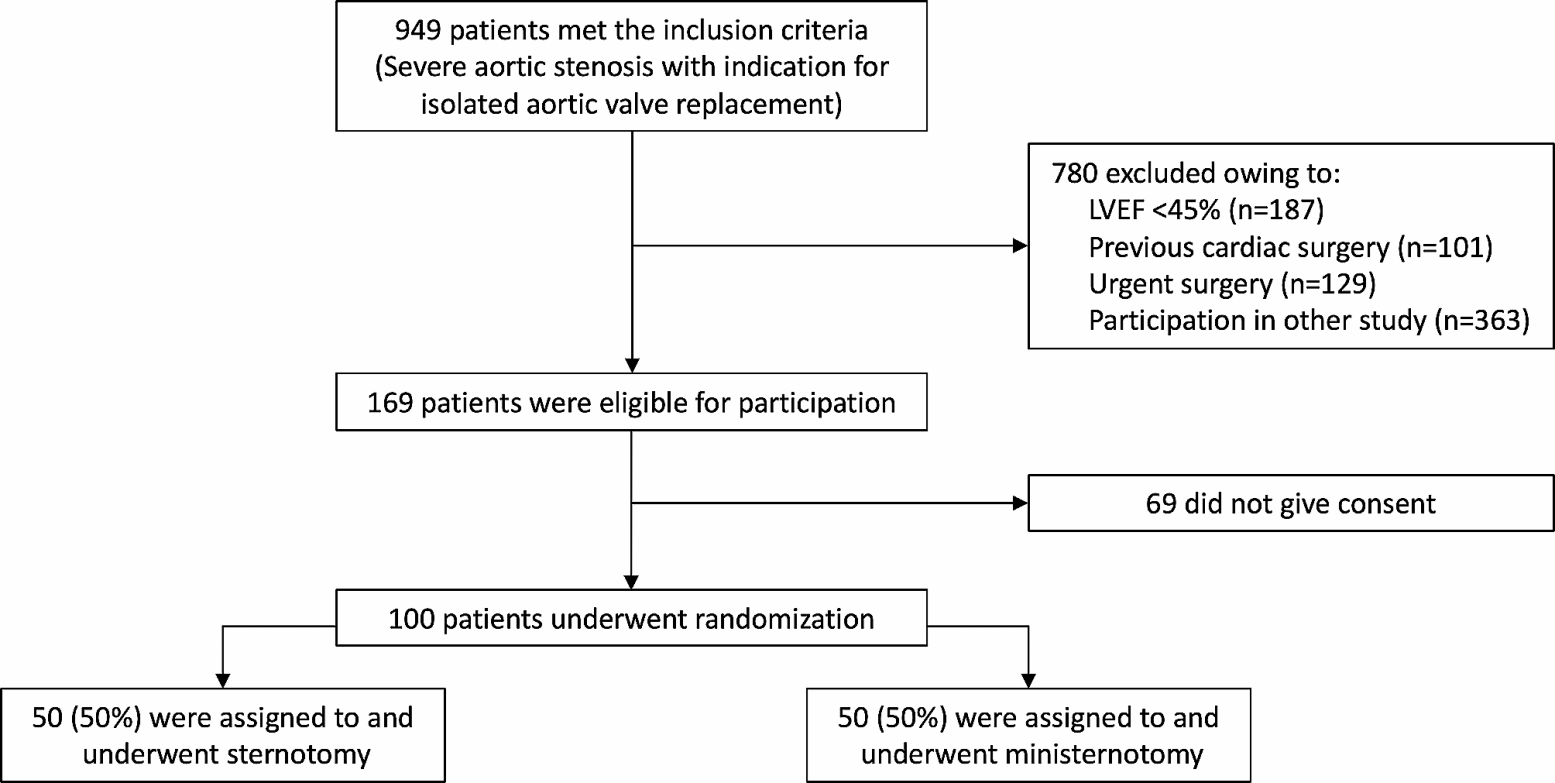
Study flow chart

Fifty patients were randomized to ministernotomy and 50 patients to full sternotomy AVR and were operated between November 2013 and August 2020 (Fig. 1). Inclusion of patients was stopped as the target number of subjects was reached, and there were no exclusions after randomization. The groups were comparable regarding baseline characteristic, including age, sex, EuroSCORE II, and preoperative antithrombotic treatment (Table 1). Operative and postoperative data are presented in Table 2. Three patients randomized to ministernotomy were intraoperatively converted to full sternotomy due to issues regarding cardioplegia administration in one case and exposure difficulties in two cases (1 peripheral venous cannula dislocated from superior vena cava to the right atrium, 1 anatomical consideration), and were analyzed in the ministernotomy group according to the intention-to-treat principle. These three patients did not suffer severe postoperative bleeding.

**Table 1:** Baseline characteristics

	Sternotomy *n* = 50	Ministernotomy *n* = 50
Age, years, mean (SD)	69.7 (6.7)	68.0 (8.5)
Female	21 (42%)	17 (34%)
Body mass index, kg/cm^2^, mean (SD)	28.0 (4.4)	26.6 (3.7)
Hypertension	34 (68%)	27 (54%)
Prior stroke	5 (10%)	1 (2%)
Diabetes mellitus	12 (24%)	9 (18%)
Chronic pulmonary disease	5 (10%)	5 (10%)
Extracardiac arteriopathy	2 (4%)	0
Atrial fibrillation	3 (6%)	2 (4%)
Prior percutaneous coronary intervention	2 (4%)	0
Poor mobility	5 (10%)	1 (2%)
Current smoker	8 (16%)	7 (14%)
Systolic pulmonary artery pressure > 30 mmHg	11 (22%)	15 (30%)
New York Heart Association class		
I	4 (8%)	0
II	22 (44%)	26 (52%)
III	24 (48%)	24 (48%)
IV	0	0
EuroSCORE II, mean (SD)	1.38 (0.66)	1.53 (1.04)
Aortic regurgitation		
None	32 (64%)	28 (56%)
Mild	11 (22%)	13 (26%)
Moderate	5 (10%)	9 (18%)
Severe	2 (4%)	0
Hemoglobin, g/l, mean (SD)	134.5 (14.4)	138.1 (14.3)
Creatinine, µmol/l, mean (SD)	79.0 (14.9)	83.8 (17.1)
Acetylsalicylic acid	13 (26%)	18 (36%)
Clopidogrel	2 (4%)	0
Warfarin	2 (4%)	2 (4%)
Non-vitamin K antagonist oral anticoagulant	1 (2%)	0

Data are n (%) unless otherwise noted. EuroSCORE II = European System for Cardiac Operative Risk Evaluation Score II

**Table 2:** Operative and postoperative data

	Sternotomy *n* = 50	Ministernotomy *n* = 50	p-value
Aortic cross-clamp time, minutes, mean (SD)	69 (19)	70 (23)	0.74
Cardiopulmonary bypass time, minutes, mean (SD)	84 (22)	96 (31)	0.02
Prosthesis type			0.48
Biological	40 (80%)	37 (74%)	
Mechanical	10 (20%)	13 (26%)	
Perioperative stroke	2 (4%)	1 (2%)	0.56
New onset atrial fibrillation	16 (32%)	17 (34%)	0.83
Permanent pacemaker implantation	2 (4%)	2 (4%)	1.00
Pneumonia treated with antibiotics	0	1 (2%)	0.31
Perioperative myocardial infarction	1 (2%)	1 (2%)	1.00
Pleurocentesis	2 (4%)	1 (2%)	0.56
Pericardiocentesis	2 (4%)	0	0.15
Maximum creatinine, µmol/l, mean (SD)	104 (48)	94 (39)	0.25
Maximum CK-MB, µg/l, mean (SD)	22 (20)	15 [12]	0.06
Superficial sternal wound infection	1 (2%)	0	0.31
Reoperation for sternal insufficiency	1 (2%)	0	0.31
Intensive care unit stay, days, mean (SD)	1.3 (0.9)	1.2 (0.4)	0.33
In-hospital stay, days, mean (SD)	5.4 (2.7)	5.4 (1.8)	0.86
Major adverse cardiac event	4 (8%)	2 (4%)	0.40
30-day mortality	3 (6%)	0	0.08

Data are n (%) unless otherwise noted. Major adverse cardiac event defined as 30-day mortality, perioperative stroke, or perioperative myocardial infarction. CK-MB = creatine kinase-myocardial band, Q = quartile, MACE = Major adverse cardiovascular events

### Bleeding-related outcomes

**Fig. 2:**
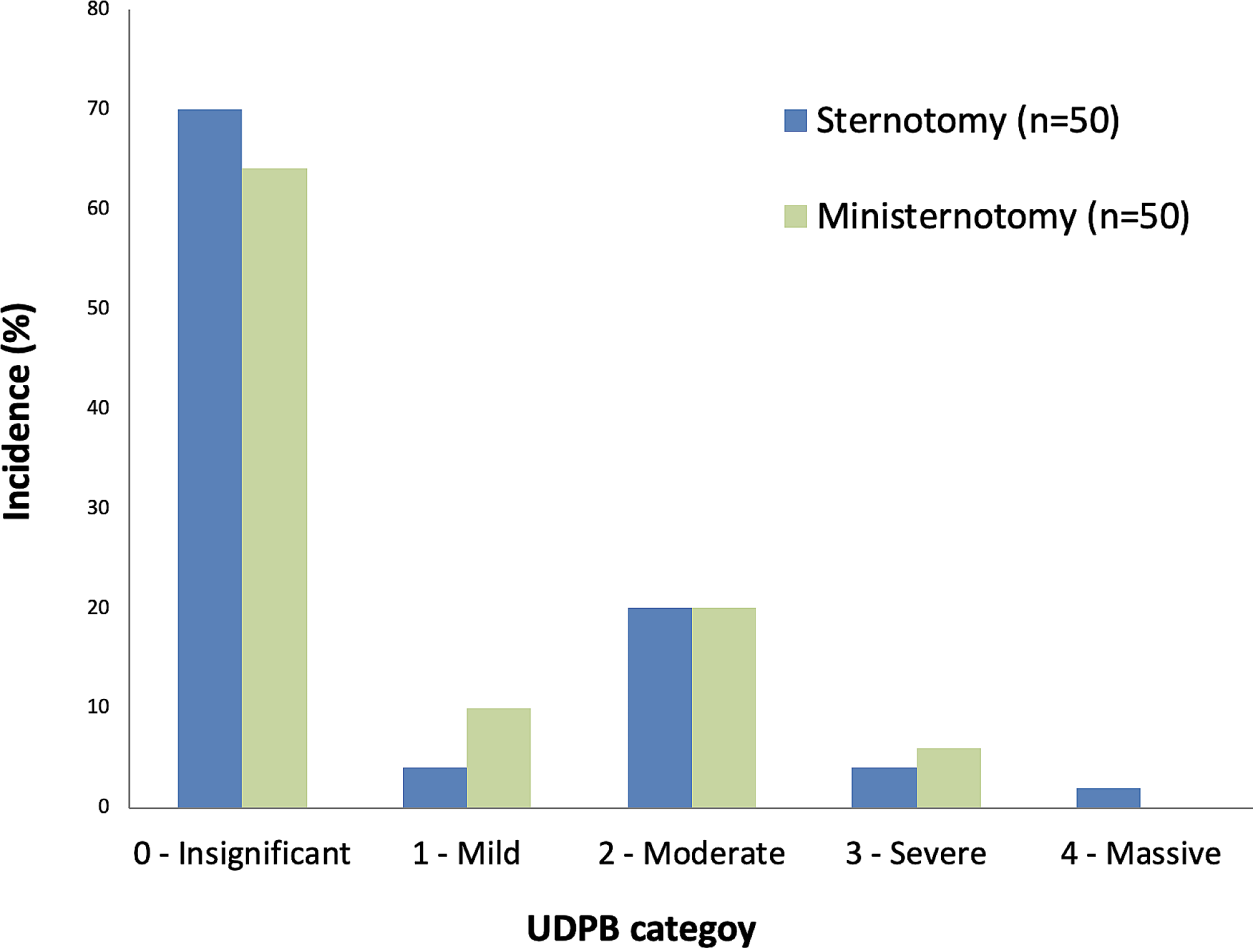
Incidence of bleeding severity among patients undergoing minimally invasive or full sternotomy aortic valve replacement, categorized according to the Universal Definition of Perioperative Bleeding (UDPB)

Three patients (6%) in each group, suffered severe or massive postoperative bleeding as defined by UDPB class 3 or 4 (Fig. 2; Table 3). Of these 6 patients, severe or massive postoperative bleeding was owing to re-exploration for bleeding in 4 cases, 12-hour chest tube output more than 1000 ml in one case, and transfusion of more than 4 packed red blood cell units in one case. Moderate bleeding was observed in 10 (20%) patients in each group. Chest tube output during the first 12 postoperative hours was mean (SD) 350 (220) ml in the full sternotomy group and 270 (190) ml in the ministernotomy group (*p* = 0.08). Transfusion with packed red blood cells were administered to 14 (28%) patients in the full sternotomy group and 18 (36%) patients in the ministernotomy group (*p* = 0.39). Transfusion rates of plasma and platelets also did not show statistically significant differences between groups. In the ministernotomy, group, both re-explorations were done via the ministernotomy, without conversion to full sternotomy. The surgical source of bleeding in the four re-explored patients were the sternum in 2, the aortotomy in 1, and unknown in 1 case.

**Table 3:** Bleeding-related outcomes

	Sternotomy *n* = 50	Ministernotomy *n* = 50	p-value	
Severe or massive UDPB category	3 (6%)	3 (6%)	1.00	
UDPB category			0.62	
0 – Insignificant	35 (70%)	32 (64%)		
1 – Mild	2 (4%)	5 (10%)		
2 – Moderate	10 (20%)	10 (20%)		
3 – Severe	2 (4%)	3 (6%)		
4 – Massive	1 (2%)	0		
Delayed sternal closure	0	0	-	
12-hour chest tube output, ml, mean (SD)	350 (220)	270 (190)	0.08	
Packed red blood cells, units, median (Q1, Q3)	0 (0, 1)	0 (0, 2)	0.50	
Any packed red blood cells transfused	14 (28%)	18 (36%)	0.39	
> 2 units of packed red blood cells	8 (16%)	6 (12%)	0.56	
Plasma, units, median (Q1, Q3)	0 (0, 0)	0 (0, 0)	0.45	
Any plasma transfused	5 (10%)	3 (6%)	0.46	
Platelet concentrates, units, median (Q1, Q3)	0 (0, 0)	0 (0, 0)	0.72	
Any platelet concentrates transfused	5 (10%)	4 (8%)	0.73	
Cryoprecipitate	0	0	-	
Prothrombin complex concentrate	0	0	-	
Recombinant activated factor VII	0	0	-	
Re-exploration for bleeding	2 (4%)	2 (4%)	1.00	

Data are n (%) unless otherwise noted. Q = quartile, UDPB = Universal definition of perioperative bleeding.

### Other outcomes

Three patients, all in the full sternotomy group, died within 30 days of surgery, and cause of death was aspiration following ileus, hemorrhagic pancreatitis, and cardiac arrest, respectively. There were no statistically significant differences between groups regarding frequency of perioperative stroke, myocardial infarction, deep sternal infection, duration of intensive care unit stay and total length of hospital stay (Table 2). Mean cardiopulmonary bypass time was 96 (31) minutes (SD) in the ministernotomy group and 84 (22) minutes in the full sternotomy group (*p* = 0.02), while aortic clamp time did not differ (Table 2).

## Discussion

This randomized controlled trial in patients with aortic stenosis did not demonstrate any advantage regarding bleeding-related outcomes with the minimally invasive approach as compared to a standard full sternotomy AVR.

The use of the UDPB definition [14] has to our knowledge not previously been reported in randomized trials comparing mini- and full sternotomy AVR. We chose a composite primary endpoint of severe bleeding because no single bleeding-related variable can be used to precisely quantify and define perioperative bleeding. Mean values for chest tube output and transfusions can be strongly affected by isolated events, while median values do not reflect the severity of major bleeding events. Using the UDPB definition, both incidence and severity of bleeding events can be simultaneously reported. Another advantage of this composite outcome variable is comparability between studies.

The two largest randomized trials comparing mini- and full sternotomy, found no improvement in their primary outcome measures transfusions and length of stay, respectively [9, 10]. The study by Hancock et al. included 270 patients and reported an identical rate of patients receiving red blood cell transfusions (17% in each group). A lower postoperative chest tube output (mean difference 145 ml) and lower non-red blood transfusions within 7 days with the ministernotomy approach was reported as a secondary outcome. The restrictive study transfusion protocol threshold was pointed out by the authors as a possible bias potentially reducing the transfusion rates in both groups. The study by Nair et al. trial, including 222 patients, found no improvement in its primary outcome length of stay. This trial also reported bleeding-related data as a secondary outcome, with no differences between groups regarding transfusions, and near identical mean 12-hour postoperative bleeding amount (323 vs. 310 ml in mini- and full sternotomy patients, respectively).

A Cochrane review of randomized trials identified a decrease in postoperative chest tube output of 158 ml (95% CI 13–303 ml), with 297 patients included in the bleeding analysis [15]. Transfusion of blood products were not analyzed. The review concluded that potential benefits in postoperative bleeding amount and length of stay were identified, suggesting the method to be a viable and effective approach. The studies included in the review were however described as small and insufficient to fully depict significant differences in bleeding complications.

The clinical significance of the difference in bleeding amount reported in the study by Hancock et al. and the Cochrane review, as well as the trend observed in the present study, is debatable, especially as there was no concurrent difference in transfusions rate or re-exploration for bleeding. Also, as pointed out in The Cochrane review, chest tube placement differs between the operation methods as the right pleural space is often opened and a pleural drain is placed after a ministernotomy, but not always after full sternotomy AVR. This could have the possible effect that a lower bleeding amount is falsely observed due to pooling of the true blood loss in the right pleural space, if not effectively reached by the chest tube. However, one can also speculate that placement of chest tube in the right pleural cavity could induce pleural irritation resulting in secretion. Our study did not analyze this difference and it should be regarded as a study limitation.

Conversion from ministernotomy to full sternotomy is somewhat a misnomer as the right sternal half remains divided, possibly causing more bleeding and rendering the sternum less stable after a conversion than after a primary full sternotomy. In the current study, intraoperative conversion rate to full sternotomy was 6% (*n* = 3), and none of them were owing to bleeding. Previous studies have shown that conversion is not a rare event, as the study by Nair et al. reported a frequency of 9.1% and the study by Hancock et al. 11.9% when conversions during re-exploration for bleeding were included. In the current study, no patient was converted to full sternotomy during re-exploration for bleeding.

### Limitations

Even though randomized, this study has some selection limitations. Randomization was done before the day of surgery and the study was not blinded. Eight consultant surgeons performed full sternotomy AVR, but only 4 of these performed both full- and ministernotomy AVR. While these four surgeons had already surmounted the learning curve for ministernotomy, the study’s time frame may have further influenced their experience with the method. The duration of the study also poses a potential risk for changes in the standard of care, although no relevant alterations in patient selection, surgical techniques, or antithrombotic treatments were introduced during the study period. Other limitations include the single institution design and the small sample size.

## Conclusions

In this study, when challenged in a randomized setting, the ministernotomy approach in AVR does not show superiority over standard full sternotomy regarding bleeding-related outcomes.

### Electronic supplementary material

Below is the link to the electronic supplementary material.

Supplementary Material 1

## Data Availability

The data underlying this article will be shared on reasonable request to the corresponding author.
